# Integrating a growth degree-days based reaction norm methodology and multi-trait modeling for genomic prediction in wheat

**DOI:** 10.3389/fpls.2022.939448

**Published:** 2022-09-02

**Authors:** Miguel Angel Raffo, Pernille Sarup, Jeppe Reitan Andersen, Jihad Orabi, Ahmed Jahoor, Just Jensen

**Affiliations:** ^1^Center for Quantitative Genetics and Genomics, Aarhus University, Tjele, Denmark; ^2^Nordic Seed A/S, Odder, Denmark; ^3^Department of Plant Breeding, Swedish University of Agricultural Sciences, Uppsala, Sweden

**Keywords:** genomic prediction, multi-trait, multi-environment, reaction norm, genotype by environment interaction

## Abstract

Multi-trait and multi-environment analyses can improve genomic prediction by exploiting between-trait correlations and genotype-by-environment interactions. In the context of reaction norm models, genotype-by-environment interactions can be described as functions of high-dimensional sets of markers and environmental covariates. However, comprehensive multi-trait reaction norm models accounting for marker × environmental covariates interactions are lacking. In this article, we propose to extend a reaction norm model incorporating genotype-by-environment interactions through (co)variance structures of markers and environmental covariates to a multi-trait reaction norm case. To do that, we propose a novel methodology for characterizing the environment at different growth stages based on growth degree-days (GDD). The proposed models were evaluated by variance components estimation and predictive performance for winter wheat grain yield and protein content in a set of 2,015 F6-lines. Cross-validation analyses were performed using leave-one-year-location-out (CV1) and leave-one-breeding-cycle-out (CV2) strategies. The modeling of genomic [SNPs] × environmental covariates interactions significantly improved predictive ability and reduced the variance inflation of predicted genetic values for grain yield and protein content in both cross-validation schemes. Trait-assisted genomic prediction was carried out for multi-trait models, and it significantly enhanced predictive ability and reduced variance inflation in all scenarios. The genotype by environment interaction modeling *via* genomic [SNPs] × environmental covariates interactions, combined with trait-assisted genomic prediction, boosted the benefits in predictive performance. The proposed multi-trait reaction norm methodology is a comprehensive approach that allows capitalizing on the benefits of multi-trait models accounting for between-trait correlations and reaction norm models exploiting high-dimensional genomic and environmental information.

## Introduction

Genomic selection (GS, Meuwissen et al., 2001) is an efficient selection method based on whole-genome prediction (WGP) that has been successfully applied for a variety of complex traits in animals and plants (De los Campos et al., 2009; Hayes and Goddard, 2010; Gianola and Rosa, 2015; Crossa et al., 2017). Over the last few decades, genomic models have been extended to accommodate greater availability of data from new technologies and increasing computational resources that allow incorporation of high dimensional data. In plant breeding, there has been an increasing interest in using multi-trait (MT) multi-environmental (ME) models over single-trait (ST) single-environmental models, as it may result in an improvement in accuracy of selection and prediction (Malosetti et al., 2007; Montesinos-Lopez et al., 2016; Montesinos-Lopez et al., 2019). MTME models combine the benefits of exploiting between-trait correlations from MT models and characterizing genotype-by-environment interactions (G×E) from ME evaluations. Efficient G×E modeling is required to counteract the negative impacts of climate change, either by selecting more resilient lines with decreased G×E or by developing lines well adapted to specific environments.

The advantages of MT models lie in that they can represent and exploit the correlation between traits, which can increase prediction accuracy and reduce bias in predictions compared to single-trait models (Henderson and Quaas, 1976; Pollak et al., 1984). The use of between-trait correlations is especially beneficial to enhance accuracy of predicted breeding values when genetic and environmental correlations are of opposite sign, and when MT models are used to infer low-heritability traits genetically correlated with high-heritability traits (Thompson and Meyer, 1986; Jia and Jannink, 2012; Jiang et al., 2015). However, MT models increase statistical complexity in the number of parameters to estimate, which could reduce the accuracy of estimation and their benefits for prediction (Haile et al., 2018; Lado et al., 2018). MT models are also useful for predicting genetic values of individuals not phenotyped for specific traits of interest but having phenotypic records for correlated traits. This may represent an additional advantage for the MT analysis, particularly if the trait of interest is difficult to measure or has high phenotyping cost.

Modeling G×E has been valuable for wheat genomic prediction (GP), as it has shown increases in prediction accuracy ranging from 10 to 40 % (reviewed by Crossa et al., 2017) and allows prediction of breeding values for lines not tested in the target environment, but genetically related with other tested lines. G×E can be identified in ME evaluations and incorporated into statistical models to detect the responses of genotypes to changing environments (also termed Macro-environmental sensitivity); the differential response of genotypes to environments is called the reaction norm (RN, Falconer, 1990; Falconer and Mackay, 1996; Calus and Veerkamp, 2003). The G×E can be modeled in an RN framework as a function of markers and environmental covariates (ECs) collected from weather stations (e.g., temperature, precipitation, solar radiation) and soil characterization (e.g., water storage capacity, sand content, hydraulic conductivity). However, given the high dimensional nature of genomic data and ECs, modeling marker × ECs interactions can lead to computational challenges.

A computationally efficient approach to quantify the G×E is using (co)variance structures. Burgueño et al. (2012) model G×E in a ME version of the genomic best linear unbiased predictor model (G-BLUP), where (co)variances structures of molecular markers and pedigree were used to represent genetic relationships within environments. The Burgueño et al. (2012) approach can also be seen as an MT methodology since environments were considered different traits, representing heterogeneous variance and covariances among environments. However, their approach did not include ECs to model G×E. Jarquin et al. (2014) developed an RN model, where the main and interaction effects of markers and ECs were introduced using high-dimensional (co)variance structures of markers and ECs. The ECs were specifically computed for different phenological stages summarizing water availability, temperature, and radiation. A similar definition of ECs has been implemented by Heslot et al. (2014), but the phenological stages were simulated using crop modeling instead of the empirical measure of phenology. Such approaches can be interpreted as RN models (Falconer, 1990; Falconer and Mackay, 1996; Calus and Veerkamp, 2003; Su et al., 2006) since phenotypes are -implicitly- linearly regressed on ECs. The RN model proposed by Jarquin et al. (2014) has been applied successfully in breeding programs of cotton and wheat (Pérez-Rodríguez et al., 2015; Crossa et al., 2016) and has recently been applied in combination with historical weather records and simulation to tackle the problem of predicting cultivars’ future performance under uncertain conditions (De Los Campos et al., 2020). Other methods have also been proposed to incorporate marker by environment interaction into the GS framework, for instance, models using Gaussian Kernel (Cuevas et al., 2016; Cuevas et al., 2017) or deep learning methodologies (Montesinos-Lopez et al., 2018) and have been successfully applied for GP.

Approaches for incorporating both MT and ME information in GS have been developed using different methodologies (Malosetti et al., 2007; Montesinos-Lopez et al., 2016; Montesinos-Lopez et al., 2019; Ward et al., 2019). However, to the best of our knowledge MT reaction norm models (MTRN) incorporating ECs information are lacking. In our study, we propose to extend a RN model incorporating G×E through high-dimensional (co)variance structures of markers and ECs (Jarquin et al., 2014; Pérez-Rodríguez et al., 2015; De Los Campos et al., 2020) to the MT case (MTRN). To do that, we propose a novel methodology for characterizing the environment at different growth stages based on growth degree-days (GDD) instead of phenological stages. Thus, GDD periods (e.g., computed each 100-GDD) encompassing the crop stages throughout the full season can be used. The GDD have been broadly recognized as one of the main forces driving phenology in wheat (Nuttonson, 1955; Slafer and Rawson, 1994; Salazar-Gutierrez et al., 2013; Aslam et al., 2017). We hypothesize that using GDD to define wheat growth periods can be useful for several reasons:

I. It simplifies the implementation of RN models since phenological records or crop simulations are no longer needed to infer the phenology of the lines. This may be convenient for wheat breeding because, in most cases, the full phenological stages are not recorded, or alternatively, it avoids the use of crop simulations that can represent a challenge for breeders since crop simulations are not commonly used in breeding programs.
II. GDD periods represent a convenient approach to deal with the differences in growth stages of breeding lines. Accurately capturing differences in the growth stages of breeding lines can be difficult using crop simulation because the critical periods for different breeding lines can occur at different moments within the same environment (year-location combinations). Nevertheless, this is no longer a problem when GDD periods are used.
III. Using GDD periods can be helpful when ECs is determined for traits where the relationships between the environmental conditions and resulting phenotype are not well established; while this may not represent a problem for wheat grain yield since these relationships have been extensively defined (Meynard and Sebillotte, 1994), it can be relevant for quality or diseases traits.

In addition, the proposed MTRN model using (co)variance structures to incorporate G×E represent a convenient choice to reduce the high demand on computational resources, which have often been a relevant restriction for developing MTME models. This study uses a large set of winter wheat breeding lines phenotyped for grain yield and protein content in multiple environments. The proposed models were evaluated for both traits using cross-validation (CV) and trait-assisted genomic prediction (TA-GP) in two prediction scenarios relevant to plant breeders: (i) predicting the performance of breeding lines that have been tested in some environments but not in others (CV1, leave-one-year-location-out), and (ii) predicting the performance of new lines across breeding cycles (CV2, leave-one-breeding-cycle-out). The TA-GP was performed considering the phenotypes of the additional trait to exploit between-trait correlations, which is intended to improve predictions of breeding values for the trait of interest.

## Materials and methods

### Plant material and phenotyping

In this study, 2,015 sixth-generation (F_6_) winter wheat lines (*T. aestivum* L.) from the breeding company Nordic Seed A/S were used; a subset of these data has been used in earlier studies (Cericola et al., 2017; Kristensen et al., 2018; Tsai et al., 2020; Raffo et al., 2022). The breeding lines came from seven breeding cycles (BC) tested in years 2014–2019, and each breeding cycle originated from approximately 60 parental line-crosses followed by five generations of selfing, including creating single seed descent (SSD) lines in generation F_4_. Each BC were composed of around 330 lines sown in one or 2 years (cycle 1: 2014, cycle 2: 2014–2015, cycle 3: 2015–2016, cycle 4: 2016–2017, cycle 5: 2017, cycle 6: 2018, cycle 7: 2019) at three locations in Denmark (DK): Odder (Central DK), Holeby (South DK), and Skive (North-west DK). For breeding cycles from 2014 to 2016 (cycle 2, 3 and 4) lines were tested in 2 years instead of one in order to have a quick construction of a reliable training population for genomic prediction. Each year the field trials were designed in 15 blocks of 46 line plots of 8.25 m^2^, having two replicates of 21 F_6_ lines and two checks randomly assigned per year-location combination. The traits analyzed were grain yield measured as kg per plot (8.25 m^2^) and protein content (%) determined by near-infrared spectroscopy (NIRS), and both traits were chosen according to their high relevance for breeding. Similar agronomic management was applied for all trials (e.g., sowing and assessment time, fertilization, application of treatments etc.).

### Genotyping

A modified CTAB method was used to perform DNA extractions (Rogers and Bendich, 1985). The plant material was genotyped using the 15K Illumina Infinium iSelect HD Custom Genotyping BeadChip technology (Wang et al., 2014). Quality control was carried out by removing genotyped SNPs with minor allele frequency (MAF) lower than 5% and call rate lower than 0.90. In total, 12,893 SNPs remained after quality control.

### Environmental data and environmental covariates

Climatic variables were collected on a daily basis and described temperature, relative humidity, wind speed, vapour-pressure deficit, and global radiation from weather stations within 20 km from field trials; precipitation within 10 km; minimum and maximum temperature and potential evaporation from the closest weather stations located in Silstrup, Askov, and Flakkebjerg for Skive, Dyngby, and Holeby, respectively (Plauborg, pers. comm.). Soil information was available in each locality for depths 0–30, 30–60, 60–100, 100–200 cm and described texture (clay, sand, silt), carbon content, hydraulic conductivity and plant available water (Adhikari et al., 2013; Kotlar et al., 2020).

**FIGURE 1:**
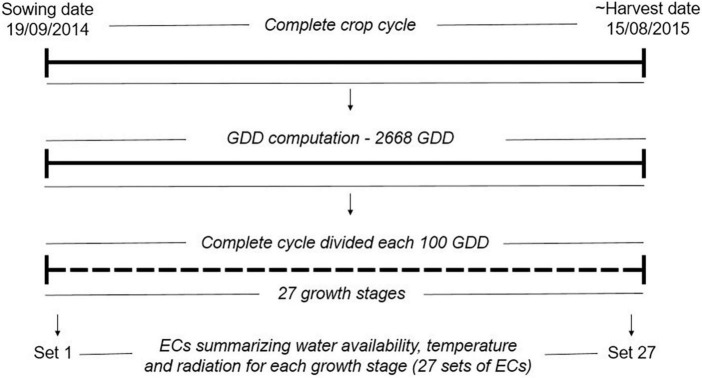
Computation of growth stages and environmental covariates (ECs) for year 2014 in the locality of Skive. In total, the complete crop cycle was divided into 27 shorter periods of 100 GDD (27 growth stages). For each growth stage, a set of ECs summarizing environmental descriptors linked to water availability, radiation and temperature were computed as described in Supplementary material 1. The same approach was used for all year-location combinations. GDD: growth degree days, the GDD were estimated as the thermal sum of daily average temperature over 0°C.

The climatic variables were used to compute ECs summarizing environmental descriptors linked to water availability, radiation, and temperature at different crop stages (see Figure 1 and Table 1), which together with the soil information, were used to describe the environments at the level of year-location. The crop stages were defined each 100 GDD, where GDD were estimated as the thermal sum of daily average temperature over 0°C (McMaster and Wilhelm, 1997; Salazar-Gutierrez et al., 2013) from sowing date until August 15 (estimated end of harvest season in Denmark). The 100-GDD stages were intended to summarize environmental conditions in short periods of days that encompass the crop phases throughout the full crop period. In total, 17 climatic variables were computed for each GDD stage, and seven soil variables specific for each locality were defined for four depths (0–30, 30–60, 60–100, 100–200 cm). Quality control of ECs was performed by removing variables with more than 10% of missing values (NA) or 30% of repeated values as indicated in Jarquin et al. (2014). After quality control, 300 climatic ECs from the first to the 27 GDD stages remained for each year-location combination plus 23 soil ECs (300 climatic ECs + 23 soil ECs = 323 ECs; see Supplementary material 1 for a complete description of variables obtained after quality control). Climatic ECs for GDD stages above 2,700 GDD did not pass quality control due to presenting more than 10% on NA values. The maturity date was simulated for the different environments following Pullens et al. (2021), and it was confirmed that all relevant growth stages until maturity were within the 27 GDD stages obtained after quality control.

**TABLE 1:** Environmental covariates (ECs) description, modified from Lecomte (2005) and Heslot et al. (2014).

Code	Variable description	Category
ave.glorad	Average global radiation (MJ/m^2^)	Radiation
ave.temp	Average temperature (°C)	Temperature
ave.vpd	Average vapour pressure deficit (VPD, kPa). VPD measures difference between air moisture and potential moisture and is related to water loss	Water/evapotranspiration
cumglorad	Accumulated global radiation (MJ/m^2^)	Radiation
ratrdtmp	Ratio between global radiation (MJ/m^2^) and temperature (°C)	Photothermal ratio
cumpospetp	Accumulated positive precipitation (mm) – evaporation (mm)	Water
cumnegpetp	Accumulated negative precipitation (mm) – evaporation (mm)	Water
cumpetp	Accumulated total precipitation (mm) – evaporation (mm)	Water
cumntdryd	Number of total dry days [precipitation (mm) ≤ evaporation (mm)]	Water
cumnsti4	Accumulated temperature (°C) lower than −4°C	Frost/temperature
cumndt0	Number of days with minimum temperature (°C) lower than 0°C	Frost/temperature
cumsti0	Accumulated temperature (°C) lower than 0°C	Frost/temperature
cumprec	Accumulated precipitation (mm)	Water
cumvpd	Accumulated vapour pressure deficit (kPa)	Water/evapotranspiration
GDD	Growth degree days estimated as the thermal sum of daily average temperature over 0°C	Temperature
ndi10m	Number of days with radiation lower to 1,045 J/cm^2^	Radiation
sri10m	Sum of daily radiation (MJ/m^2^) when radiation is lower to 1,045 J/cm^2^	Radiation
pvt	Plant available water (%)	Soil/water
wscmm	Water storage capacity (mm)	Soil/water
claynor	Sand content (%)	Soil
fsandno	Fine sand content (%)	Soil
gsandno	Coarse sand content (%)	Soil
kulstof	Carbon content (%)	Soil
siltnor	Silt content (%)	Soil
Ks_250	Saturated hydraulic conductivity (%)	Soil

The ECs for the categories radiation, temperature, frost, photothermal ratio, water and evapotranspiration were computed each 100 GDD for the complete growth cycle starting from the sowing date. The ECs for the category soil were available at all localities for four depths: 0–30, 30–60, 60–100, 100–200 cm. The ECs from the different categories were used together to describe the environmental conditions in each year-location combination.

### Statistical analysis

In this study, five models were proposed to evaluate the effect of including G×E interaction and MT modeling for grain yield and protein content. The full data set was used for VCs estimation to obtain estimates as accurate as possible. First, a baseline mixed model without genomic information (M1) was used as a starting point for constructing the other models; a similar baseline model has been used in earlier works with Nordic Seed A/S data (Cericola et al., 2017; Tsai et al., 2020). Second, the baseline model was extended by a genomic [SNPs] effect (M2), capturing the main additive genetic effects (Habier et al., 2007; VanRaden, 2008). Third, a G×E effect based on genomic [SNPs] by ECs interactions extended M2 to the RN framework (M3). Lastly, M2 and M3 were extended to the MT case (M4 and M5, respectively), considering grain yield and protein content. A detailed model description is provided below.

#### Model 1 (M1, baseline model)

**FIGURE 2:**
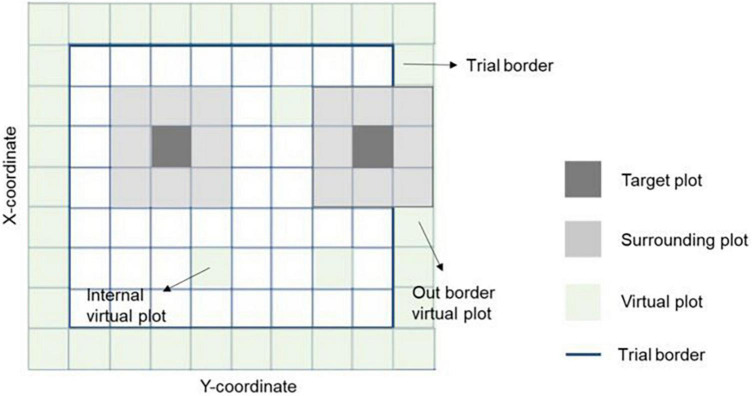
Representation of spatial information in a field trial. The target and eight surrounding plots were used together to correct the spatial variability across the field. The trial borders’ effect was considered by adding virtual plots to complete the eight surrounding plots for all observations. Virtual plots were also added in empty X-Y coordinates (with no plot observation registered) to ensure all plots have the eight surrounding plots. Hence, the spatial effects on an individual plot is the sum of effects with the square centered on the plot itself plus the effects of the eight surrounding plots with a square centered on those plots.

**FIGURE 3:**
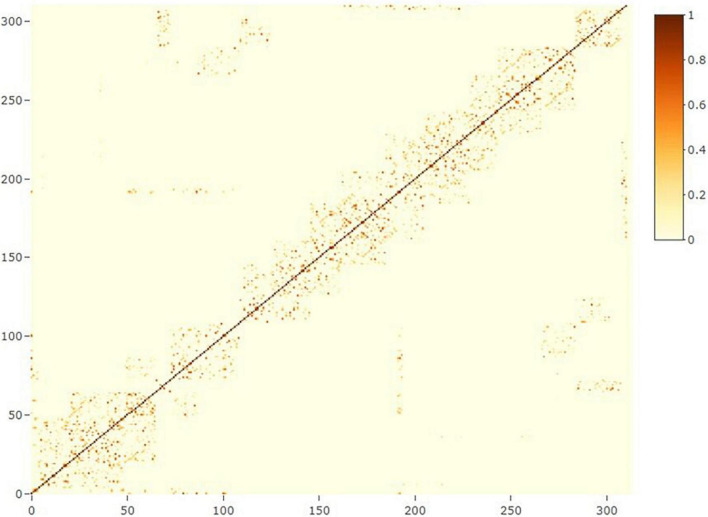
Heatmap of the spatial relationship matrix for locality Dyngby in the year 2017. A total of 311 plots were observed at Dyngby 2017, and the spatial relationship between plots are represented. Higher to lower relationships are represented from yellow to light-blue colors; the dark blue represents a lack of relationship between plots (not neighboring connections).

The M1 (Equation 1) is a mixed model considering the main sources of variability affecting the data and the experimental design. It was defined as:


(1) $$y_{n}=X\,b+Z_{1}\,l+Z_{2}\,f+Z_{3}\,s+e$$

where **y_n_** is the vector of phenotypes for grain yield (**n** = 1) or protein content (**n** = 2); **X** and **Z_n_**(**n** = 1, 2, 3) are design matrices for fixed and random effects, respectively; **b** is the vector of fixed trial effects nested within year-location-breeding cycle, which is intended to capture the variation due to overall effects of year, location, the interaction between year and location as well as effects of the spatial location of the trial within the field.; **l** is a vector of line effect with $\text{l}∼N\,I\,I\,D\,\left(0,\text{I}\,\sigma_{l}^{2}\right)$, where **I** is an identity matrix and $\sigma_{l}^{2}$ is the variance due to uncorrelated line effects; **f** is a vector of line × environment interaction (L×E) with $\text{f}∼N\,I\,I\,D\,\left(0,\text{I}\,\sigma_{f}^{2}\right)$, where $\sigma_{f}^{2}$ is the variance due to uncorrelated L×E effects; **s** is a vector of spatial effect, which follows a multivariate normal density (MVN) with $\text{s}∼M\,V\,N\,\left(0,\text{S}\,\sigma_{s}^{2}\right)$, where **S** is a spatial relationship matrix and $\sigma_{s}^{2}$ is the spatial effect variance. The spatial effect was defined as the combination of 9-spatial sub-components, where one sub-component is the spatial effect for the square centered on the plot of the observation (i.e., target plot), and the eight remaining sub-components are the spatial effects for the square centered on the eight plots surrounding the target plot (Figure 2). Virtual plots were added over trial borders and into empty spaces of the X by Y grid to control border effects and ensure all plots have eight surrounding plots. The spatial effects were identified by assigning a unique id given by its X (row) and Y (column) coordinates. The spatial relationship matrix **S** was computed as $\text{S}=\frac{{\mathrm{XX}}^{\prime }}{t\,r\,\left({\mathrm{XX}}^{\prime }\right)/n}$, where X_n× q_ is a *n* × *q* matrix, with *n* = number of observations (i.e., number of real plots), and *q* = number of real plus virtual plots. The X_n× q_ is an indicator matrix relating observations to spatial effects in **S**. A heatmap of the **S** matrix for a subset of the data (Dyngby, 2017) is shown in Figure 3. Additional explanations for the modeling of the spatial effect can be found in Tsai et al. (2020), where the spatial effect was equivalently modeled by the first time, but with the difference that for our case we used the relationship matrix **S**, and they used the regression of the 9-spatial covariates for each plot observation directly as random effects. However, although these two implementations are mathematically equivalent, our implementation is computationally faster. The **e** is a vector of random residuals with $\text{e}∼N\,I\,I\,D\,\left(0,\text{I}\,\sigma_{e}^{2}\right)$, where $\sigma_{e}^{2}$ is the residual variance.

#### Model 2 (M2)

The M2 (Equation 2) extend M1 by adding a genomic [SNP] additive effect using the VanRaden (2008) genomic relationship matrix as (co)variance structure, and therefore, M2 can be seen as a G-BLUP model (Habier et al., 2007; VanRaden, 2008). M2 was defined as:


(2) $${\text{y}}_{n}=\text{X}\,b+{\text{Z}}_{1}\,\text{l}+{\text{Z}}_{2}\,\text{f}+{\text{Z}}_{3}\,\text{s}+{\text{Z}}_{4}\,\text{g}+\text{e}$$

where **y_n_**, **X**, **Z_n_**, **b**, **l**, **f**, **s**, and **e** were defined as in M1; **g** is a vector of the genomic additive breeding values (GEBVs), with $\text{g}∼M\,V\,N\,\left(0,\text{G}\,\sigma_{g}^{2}\right)$, $\sigma_{g}^{2}$ is the genomic additive variance, and **G** is the genomic relationship matrix (GRM) based on the first method proposed by VanRaden (2008): $\text{G}=\frac{{\mathrm{ZZ}}^{\prime }}{2\,\sum p_{i}\,\left(1-p_{i}\right)}$, where **p_i_** is the minor allele frequency (MAF) of the *i^th^SNP*;**Z** is **M**−**P**; **M** is a SNP matrix coded −1, 0, 1, and **P** is a matrix with the MAF of SNP *i* calculated as **1**(2(*p*_*i*_−0.5)) for column *i*.

The line effect (**l**) was kept in all genomic models since it is intended to capture non-additive variance and additive variance not captured by SNPs in the genomic effect; therefore, this model definition helps to improve the characterization for the specific additive genomic variance captured by the SNPs.

The narrow (*h*^2^) and broad-sense (*H*^2^) plot heritabilities were estimated for M2 as $h^{2}=d\,\left(G\right)\,\sigma_{g}^{2}/\sigma_{P}^{2}$ and $H^{2}=\left(\sigma_{l}^{2}+d\,\left(G\right)\,\sigma_{g}^{2}\right)/\sigma_{P}^{2}$, where *d*(**G**) is the mean diagonal value of **G** with *d*(**G**) = 1.869, the *d*(**G**) value can be interpreted as 1 plus the average genomic inbreeding coefficient for the population (VanRaden, 2008); $\sigma_{g}^{2}$ and $\sigma_{l}^{2}$ are the genomic estimated additive and line variance as defined for M2 and M1, respectively; $\sigma_{P}^{2}$ is the estimated phenotypic variance of the plot calculated as: $\sigma_{P}^{2}=\sigma_{l}^{2}+d\,\left(G\right)\,\sigma_{g}^{2}+\sigma_{f}^{2}+\sigma_{s}^{2}+\sigma_{e}^{2}$, with $\sigma_{l}^{2}$, *d*(**G**), $\sigma_{g}^{2}$, $\sigma_{f}^{2}$, $\sigma_{s}^{2}$, and $\sigma_{e}^{2}$ as defined previously in M2 and M1. Note that *h*^2^ and *H*^2^ were reported at the plot level and not for family means as sometimes are used in plant breeding.

#### Model 3 (M3)

The M3 (Equation 3) is the most developed model in terms of definition of effects as it extends M2 by including a genomic [SNPs] × ECs interaction effect (**gw**), and therefore, it can be viewed as a linear RN model where the genetic and environmental gradients are specified as regressions on markers and ECs. As pointed in Jarquin et al. (2014) and Pérez-Rodríguez et al. (2015), the M3 uses the (co)variance patterns induced by linear-by-linear reaction norm, where the intercept of the reaction norm are implicitly in the main effects of lines and environment and the slope in the genomic [SNPs] × ECs interaction effect. The M3 was defined as:


(3) $${\text{y}}_{n}=\text{X}\,b+{\text{Z}}_{1}\,\text{l}+{\text{Z}}_{2}\,\text{f}+{\text{Z}}_{3}\,\text{s}+{\text{Z}}_{4}\,\text{g}+{\text{Z}}_{5}\,\text{g}\,w+\text{e}$$

where **y_n_**, **X**, **Z_n_**, **b**, **l**, **f**, **s**, **g**, and **e** were defined as in M1 and M2; **gw** is a vector of genomic [SNPs] additive × ECs interaction values with $\text{gw}∼M\,V\,N\,\left(0,\left({\text{Z}}_{g}\,{\text{GZ}}_{g}^{\prime }\right){}^{\circ}\Omega \,\sigma_{g\,w}^{2}\right)$, where **Z_g_** is a design matrix connecting phenotypic observations with cultivars, **G** is the GRM, and**Ω** is the (co)variance matrix computed as in De Los Campos et al. (2020) and derived from ECs as $\Omega =\frac{{\mathrm{WW}}^{\prime }}{q}$, where W_nxq_ is a matrix of centered and scaled ECs with *n* rows (number of phenotypic observations) and *q* columns (number of ECs). The operator “°” represents the cell-by-cell (or Hadamard) product between matrices, and $\sigma_{g\,w}^{2}$ is the variance term associated with the genomic [SNPs] × ECs interaction effect.

Note that the inclusion of the line × environment interaction (**f**) and the genomic [SNPs] × ECs interaction effects together in M3 is justified since ECs do not capture all the environmental variability, and therefore, this definition of interactions with the environment contribute to specifying what is not captured by ECs. A similar concept is also extended to the main line effect (L) effect, which are present in all the defied models and are intended to cover the misspecification of the SNPs on genetic effects. The inclusion of an additional model term defining the main effect of ECs have also been used in previous works (Jarquin et al., 2014; Pérez-Rodríguez et al., 2015; De Los Campos et al., 2020); however, such an effect is not possible to estimate in our case since the main environmental effect is implicitly defined and estimated by the fixed effects of our models.

All models presented so far have been implemented in a single-trait (ST) approach for grain yield and protein content. In the following section we summarize the extension of ST models to the MT case.

#### Multi–trait models (M4-M5)

The M2 and M3 were extended to the MT case by M4 and M5 (Equation 4), respectively. The MT models considered grain yield and protein content in a bivariate analysis. The MT methodology allows exploiting between traits (co)variances for the different model effects, which modified the definition of the random effects from ST models. Following, we present the definition of the effects for the M5 (the most developed model in terms of effects and traits included):


$$\left[\begin{array}{c} y_{1}\\ y_{2} \end{array}\right]=\left[\begin{array}{cc} X_{1} & 0\\ 0 & X_{2} \end{array}\right]\,\left[\begin{array}{c} b_{1}\\ b_{2} \end{array}\right]+\left[\begin{array}{cc} Z_{1} & 0\\ 0 & Z_{2} \end{array}\right]\,\left[\begin{array}{c} l_{1}\\ l_{2} \end{array}\right]+\left[\begin{array}{cc} Z_{3} & 0\\ 0 & Z_{4} \end{array}\right]\,\left[\begin{array}{c} f_{1}\\ f_{2} \end{array}\right]$$

$$+\left[\begin{array}{cc} Z_{5} & 0\\ 0 & Z_{6} \end{array}\right]\,\left[\begin{array}{c} s_{1}\\ s_{2} \end{array}\right]+\left[\begin{array}{cc} Z_{7} & 0\\ 0 & Z_{8} \end{array}\right]\,\left[\begin{array}{c} g_{1}\\ g_{2} \end{array}\right]$$

(4) $$+\left[\begin{array}{cc} Z_{9} & 0\\ 0 & Z_{10} \end{array}\right]\,\left[\begin{array}{c} g\,w_{1}\\ g\,w_{2} \end{array}\right]+\left[\begin{array}{c} e_{1}\\ e_{2} \end{array}\right]$$

where **y_n_**, **X**, **Z_n_**, **b**, **l**, **f**, **s**, ***g, gw*** are the same as defined in the previous models for grain yield and protein content, except for the random effect variances which under the MT framework becomes: $\left[\begin{array}{l} l_{1}\\ l_{2} \end{array}\right]$ ∼*NIID*(0,**I**⊗**L**), where ⊗ denotes the Kronecker product, and **L** the line (co)variance matrix $\left[\begin{array}{l} \sigma_{l1}^{2}\;\sigma_{l12}^{2}\\ \;\sigma_{l21}^{2}\;\sigma_{l2}^{2} \end{array}\right];\;\left[\begin{array}{l} f_{1}\\ f_{2} \end{array}\right]$ ∼*NIID*(0,**I**⊗**F**) with **F** the line × environment (co)variance matrix $\left[\begin{array}{c} \sigma_{f\,1}^{2}\,\sigma_{f\,12}^{2}\\ \sigma_{f\,21}^{2}\,\sigma_{f\,2}^{2} \end{array}\right]$; $\left[\begin{array}{l} s_{1}\\ s_{2} \end{array}\right]$ ∼*MVN*(0,**S**⊗**H**) with **H** the spatial (co)variance matrix $\left[\begin{array}{c} \sigma_{s\,1}^{2}\,\sigma_{s\,12}^{2}\\ \sigma_{s\,12}^{2}\,\sigma_{s\,2}^{2} \end{array}\right]$; $\left[\begin{array}{l} g_{1}\\ g_{2} \end{array}\right]$ ∼*MVN*(0,**G**⊗**K**) with **K** the genomic (co)variance matrix $\left[\begin{array}{c} \sigma_{g\,1}^{2}\,\sigma_{g\,12}^{2}\\ \sigma_{g\,12}^{2}\,\sigma_{g\,2}^{2} \end{array}\right]$; $\left[\begin{array}{c} g\,w_{1}\\ g\,w_{2} \end{array}\right]∼M\,V\,N\,\left(0,\left({\text{Z}}_{g}\,{\text{GZ}}_{g}^{{}^{\prime }}\right){}^{\circ}\Omega \,⊗\text{P}\right)$ with **P** the genomic [SNPs] × ECs (co)variance matrix $\left[\begin{array}{c} \sigma_{g\,w\,1}^{2}\,\sigma_{g\,w\,12}^{2}\\ \sigma_{g\,w\,12}^{2}\,\sigma_{g\,w\,2}^{2} \end{array}\right]$, and $\left[\begin{array}{l} e_{1}\\ e_{2} \end{array}\right]$ ∼*NIID*(0,**I**⊗**R**) with **R** the residual (co)variance matrix $\left[\begin{array}{c} \sigma_{e\,1}^{2}\,\sigma_{e\,12}^{2}\\ \sigma_{e\,21}^{2}\,\sigma_{e\,2}^{2} \end{array}\right]$. Moreover, for MT models (M4-M5), we calculated the between-trait correlation for all model effects; for example, the genetic correlation was: $C\,O\,V_{g\,12}/\sqrt{\sigma_{g\,11}^{2}\,\sigma_{g\,22}^{2}}$, where *COV*_*g*12_ is the between-trait covariance for the genomic effect, and $\sigma_{g\,11}^{2}$ and $\sigma_{g\,22}^{2}$ are the variances associated to grain yield and protein content, respectively.

A summary of the effects included in the models and the ST or MT case is presented in Table 2. All proposed models were developed under the context of a multivariate normal distribution assuming Gaussian priors for random effects and the analyses were performed using the BGLR R package (Pérez and de los Campos, 2014) with 50,000 iterations, burn-in of 10,000, and a thinning of 10. The initial specifications in BGLR were set to an R^2^ = 0.90 to approximate the R^2^ expected of our models (known from previous data analysis using Average Information Restricted Maximum Likelihood, Cericola et al., 2017; Raffo et al., 2022) and degree of freedom (df) = 0.0001 to set uninformative priors. The posterior standard deviation (PSD) for VCs estimates was computed. In addition, the convergence on parameter estimation was analyzed using the package CODA in R (R Core Team, 2014) by estimating the Monte Carlo standard errors (MCMC error), effective sample (ESS) and by graphical analysis of Markov chains trace plots and posterior density plot. The convergence analysis is presented in Supplementary material 2.

**TABLE 2:** Summary of the effect included in the models and single-trait (ST) or multi-trait (MT) case.

Models	Main effect			Interactions		ST/MT
	*l*	*g*	*s*	*f*	*gw*	
M1 (baseline)	**×**		**×**	**×**		ST
M2	**×**	**×**	**×**	**×**		ST
M3	**×**	**×**	**×**	**×**	**×**	ST
M4	**×**	**×**	**×**	**×**		MT
M5	**×**	**×**	**×**	**×**	**×**	MT

*l* line, *g* genomic [SNPs] additive effect, *s* spatial effect, f line × environment interaction, *gw* genomic [SNPs] additive × ECs interaction. ST, single trait model; MT, multi-trait model.

### Cross-validation analysis and model validation

The predictive performance of the proposed models was evaluated using two CV approaches. The CV1 (leave-one-year-location-out) was carried out by masking the phenotypes of one year-location in the validation set and using the remaining phenotyped lines to predict the masked lines (Supplementary material 3). This process was repeated n times (n = number of year-locations = 17) until predictions for all year-locations were obtained. The CV1 simulates the prediction problem where breeding lines have been tested in some environments but not in others, and the genetic values for lines in the untested environment are desired. The CV2 (leave-one-breeding-cycle-out) was carried out by masking the phenotypes of one BC in the validation set and using the remaining phenotyped lines to predict the masked lines (Supplementary material 3). This process was repeated n-times (n = no. of breeding cycles = 7) until all BCs were predicted. The CV2 simulates the prediction problem where a new generation (newly developed lines) is predicted from parental and historical records. In addition, the MT models (M4-M5) were evaluated using TA-GP, where the phenotypic data in CV1 and CV2 was masked only for one of the traits in the validation set, and the phenotypes for the second trait were available for all lines.

The predictive ability (PA, $r_{\hat{g}\;,p}$) of the models was calculated as the Pearson correlation between the average value of lines in each year-location after correcting by fixed effects and the vector of predictions [$\rho \,\left(\bar{\text{y}},\hat{{\text{g}}_{g}}\right)$ and $\rho \,\left(\bar{{\text{y}}_{c}},\hat{{\text{g}}_{g}}+\hat{{\text{g}}_{g\,w}}\right)$]. The fixed effects were estimated for each model using the full dataset in order to obtain as accurate estimates as possible. The lines corrected by fixed effects were computed subtracting the fixed effects from each corresponding plot observation, and averaging the resulting lines values within year-locations. The PA was obtained for predictions of the main additive effect [$\rho \,\left(\bar{{\text{y}}_{c}},\hat{{\text{g}}_{g}}\right)$] of M2, M3, M4, and M5, and for predictions of the main additive effect plus the genomic [SNPs] × ECs interactions effect [$\rho \,\left(\bar{{\text{y}}_{c}},\hat{{\text{g}}_{g}}+\hat{{\text{g}}_{g\,w}}\right)$] for M3 and M5. We used an ordinary non-parametric bootstrap with replacement based on full sample size (n = 2,015), and 10,000 replicates to obtain PA standard errors. The PA between models was contrasted using a two-tailed paired *t*-test (critical *P*-value = 0.01). The maximum PA was calculated for **g** and **g** + **g*w*** predictions in the different models; for M2, M4 it was calculated as $\sqrt{\text{n}\,h_{f}^{2}/\left(1+\left(n-1\right)\,h_{f}^{2}\right)}$, where *n* was the average number of lines repetitions within year-location, $h_{f}^{2}$ was the family-based heritability and hence is affected by the experimental design (number of replications). For M3 and M5 it was calculated using the same formula but substituting $h_{f}^{2}$ with the proportion of total variance explained by the genomic additive plus the genomic interaction effects (genomic [SNPs] × ECs interaction).

A test for variance inflation in the predicted genetic effects was performed as the slope of the regression of predicted values obtained with whole information (subscript *w*, estimations with complete phenotypic information for all genotypes) on the estimated with partial information (subscript *p*, predictions for all genotypes from CVs when their phenotypes were masked): $b_{w,p}=\frac{c\,o\,v\,\left(\hat{{\text{u}}_{w}}\;\hat{{\text{u}}_{p}}\right)}{v\,a\,r\,\left(\hat{{\text{u}}_{p}}\right)}$ (Legarra and Reverter, 2018).

## Results

### Phenotyping

The descriptive statistics for grain yield and protein content are shown in Table 3. In total, 2,015 lines and 14,430 plot observations were obtained, and normal distribution was observed for both traits. Grain yield had an average value of 8.85 kg grain/8.25 m^2^ with a coefficient of variation of 11.63%. Protein content had an average value of 9.84%, with a coefficient of variation of 8.11%. The between traits correlation based on the observed phenotypes was in general negative for the different breeding cycles and had an average of −0.18 (Table 3). The variance for descriptive statistics was 1.06 for grain yield and 0.64 for protein content.

**TABLE 3:** Descriptive statistics for the grain yield and protein content of F6 wheat breeding lines.

Breeding cycle	No. of lines*	No. of plots	Trait**	Average (SD)	Min.–Max. values	Coef. of var. (%)
1	321	1,274	Yield	8.82 (0.83)	3.85–11.00	9.51
			Protein	9.67 (0.93)	7.50–15.10	9.65
2	230	2,258	Yield	8.61 (1.09)	4.75–11.47	12.70
			Protein	9.74 (0.87)	7.50–14.30	8.95
3	336	3,289	Yield	8.31 (1.11)	5.03–11.80	13.40
			Protein	10.25 (0.69)	8.40–13.00	6.73
4	159	918	Yield	9.09 (0.98)	6.21–11.40	10.78
			Protein	10.67 (0.57)	9.00–12.60	5.34
5	358	1,674	Yield	8.59 (0.46)	7.06–10.25	5.35
			Protein	8.84 (0.41)	7.60–10.20	4.46
6	257	1,977	Yield	9.36 (1.10)	6.04–12.36	11.78
			Protein	9.94 (0.51)	8.50–12.00	5.13
7	354	3,040	Yield	9.37 (0.68)	6.24–11.54	7.27
			Protein	9.73 (0.59)	8.40–12.50	6.04
Total	2,015	14,430	Yield	8.85 (1.03)	3.85–12.35	11.63
			Protein	9.84 (0.80)	7.50–15.10	8.11

*The values presented correspond to the obtained F_6_ populations after successful phenotyping and genotyping.

**Units of measure: yield (grain yield, kg grain/8.25 m^2^), protein content (%); No., number; SD, standard deviation; Min, Minimum; Max, Maximum; Coef. of var., Coefficient of variation.

### Variance components, heritability and trait correlations

Five proposed models differing in the incorporation (M2–M5) or not (M1) of genomic information, the incorporation of genomic [SNPs] × ECs interaction (M3 and M5), and the single-trait (M1, M2 and M3) or multi-trait case (M4 and M5) were used to estimate VCs for grain yield (Table 4) and protein content (Table 5).

**TABLE 4:** Posterior mean of variance components for grain yield (kg grain/8.25 m^2^).

Models	Main effect			Interactions		Res.
	*l*	*g*	*s*	*f*	*gw*	
M1 (baseline)	0.093 (0.005)*		0.067 (0.003)	0.122 (0.002)		0.055 (0.002)
M2	0.049 (0.004)	0.060 (0.008)	0.067 (0.002)	0.122 (0.002)		0.055 (0.001)
M3	0.050 (0.004)	0.040 (0.008)	0.065 (0.002)	0.047 (0.002)	0.112 (0.008)	0.056 (0.002)
M4	0.051 (0.004)	0.064 (0.008)	0.067 (0.002)	0.122 (0.002)		0.056 (0.002)
M5	0.051 (0.004)	0.049 (0.007)	0.065 (0.002)	0.048 (0.003)	0.112 (0.008)	0.056 (0.002)

*The values between parentheses are the posterior standard deviation (PSD) of the estimates.

*l* line, *g* genomic [SNPs] additive effect, *s* spatial effect, *f* line × environment interaction, *gw* genomic [SNPs] additive × ECs interaction, Res. residuals.

**TABLE 5:** Posterior mean of variance components for protein content (%).

Models	Main effect			Interactions		Res.
	*l*	*g*	*s*	*f*	*gw*	
M1 (baseline)	0.076 (0.004)*		0.050 (0.002)	0.044 (0.002)		0.049 (0.001)
M2	0.032 (0.004)	0.069 (0.008)	0.050 (0.002)	0.044 (0.002)		0.049 (0.001)
M3	0.032 (0.004)	0.058 (0.007)	0.050 (0.002)	0.018 (0.002)	0.037 (0.003)	0.048 (0.001)
M4	0.033 (0.004)	0.067 (0.007)	0.050 (0.002)	0.045 (0.002)		0.048 (0.001)
M5	0.032 (0.004)	0.058 (0.007)	0.050 (0.002)	0.020 (0.002)	0.039 (0.003)	0.047 (0.001)

*The values between parentheses are the posterior standard deviation (PSD) of the estimates.

*l* line, *g* genomic [SNPs] additive effect, *s* spatial effect, *f* line × environment interaction, *gw* genomic [SNPs] additive **×** ECs interaction, Res. residuals.

The phenotypic variances ($\sigma_{P}^{2}$) for the different models represented a proportion of 31.9–35.8 % and 34.1–38.4 % of the descriptive statistical variance for grain yield and protein content, respectively. The difference between the variance from descriptive statistics and $\sigma_{P}^{2}$ can be attributed to the considerable amount of variation captured by the fixed effects. The M1 (model without genomic information) captured lower variance related to the main genetic effect than models including the additive genomic [SNPs] term. For example, comparing line variance from M1 to line plus genomic variance from M2, the M2 captured around 17 and 33 % more genetic variance for grain yield and protein content, respectively; this is attributed to correctly accounting for covariances among lines when the additive genomic [SNPs] effect is included in the models. The narrow- and broad-sense heritabilities at plot level were estimated using M2, and higher values were observed for protein content: *h*^2^ = 0.282 (PSD = 0.025) and *H*^2^ = 0.414 (PSD = 0.017) than for grain yield: *h*^2^ = 0.168 (PSD = 0.020), *H*^2^ = 0.307 (PSD = 0.153). The G×E variability was accounted for the line × environment (**f**) and genomic [SNPs] × ECs interaction (**g*w***) effects, and a higher amount of G×E variance was observed for grain yield (~40% of $\sigma_{P}^{2}$) than for protein content (~ 17% of $\sigma_{P}^{2}$).

The VCs estimates for single-trait models M2 and M4 were similar to MT models M3 and M5, respectively. The between-trait correlations were analyzed for all terms in M3 and M5 (Table 6), and negative correlations were observed for all the effects. High negative between-trait correlations (>0.40) were observed for the main genetic model effects [line (**l**), additive (**g**)], and the interaction effects (line × environment (**f**), and genomic [SNPs] × ECs interaction (*gw*).

**TABLE 6:** Between-trait correlations for model effects of multi-trait models (M4 and M5).

Models	Main effect			Interactions		Res.
	*l*	*g*	*s*	*f*	*gw*	
M4	−0.500 (0.066)*	−0.404 (0.049)	−0.252 (0.027)	−0.687 (0.029)		−0.019 (0.019)
M5	−0.536 (0.063)	−0.419 (0.058)	−0.252 (0.027)	−0.597 (0.057)	−0.677 (0.032)	−0.016 (0.019)

*The values between parentheses are the posterior standard deviation (PSD) of covariances estimates.

*l* line, *g* genomic [SNPs] additive effect, *s* spatial effect, *f* line × environment interaction, *gw* genomic [SNPs] additive **×** ECs interaction, Res. residuals.

The MCMC errors, effective sample (ESS), and graphical analysis of trace and posterior density plots were performed as control of convergence for VCs estimates (Supplementary material 2). The parameters estimated for all models had good convergence as revealed by the MCMC error in the order of 1 × 10^–4^ or lower, the high ESS, and the appropriate trace and posterior density plots.

### Predictive ability of genomic predictions

**FIGURE 4:**
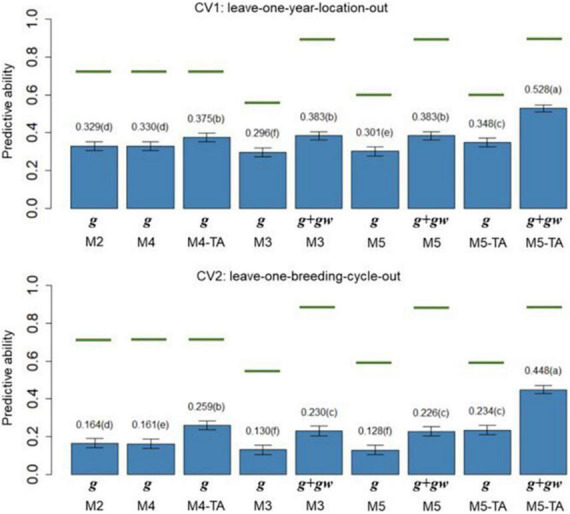
Barplot of predictive abilities (PAs) for grain yield in leave-one-year-location-out (CV1, *upper panel*) and leave-one-breeding-cycle-out (CV2, *lower panel*) cross-validations. M2: line + genomic [SNPs] additive effect + spatial effect + line × environment interaction. M3 expand M2 by adding a genomic [SNPs] additive × ECs interaction. M4 expand M2 to the multi-trait case. M4-TA is the M4 using trait-assisted (TA) genomic prediction. M5 expand M3 to the multi-trait case. M5-TA is the M4 using TA genomic prediction. Black bars are the 95% confidence interval. Differences in the letter above the bar represent significant differences between models (*P-value* < 0.01). Green lines are the theoretical maximum PAs.

**FIGURE 5:**
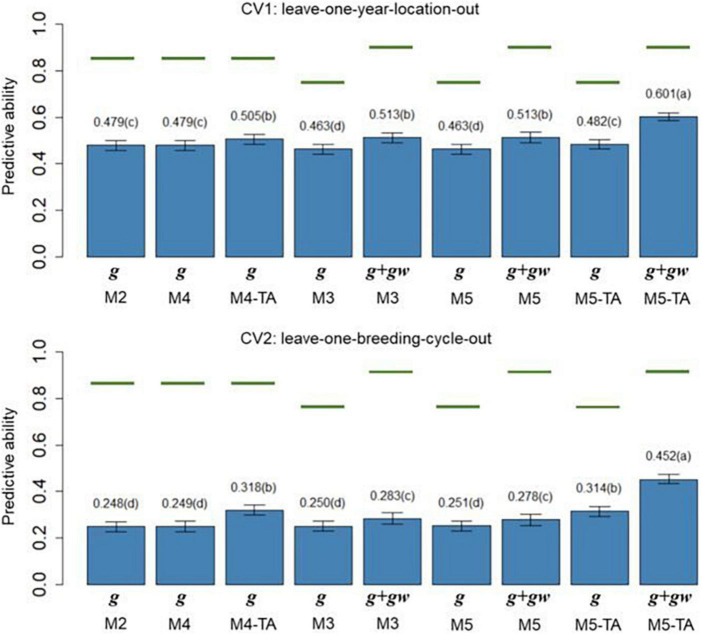
Barplot of predictive abilities (PAs) for protein content in leave-one-year-location-out (CV1, *upper panel*) and leave-one-breeding-cycle-out (CV2, *lower panel*) cross-validations. M2: line + genomic [SNPs] additive effect + spatial effect + line × environment interaction. M3 expand M2 by adding a genomic [SNPs] additive × ECs interaction. M4 expand M2 to the multi-trait case. M4-TA is the M4 using trait-assisted (TA) genomic prediction. M5 expand M3 to the multi-trait case. M5-TA is the M4 using TA genomic prediction. Black bars are the 95% confidence interval. Differences in the letter above the bar represent significant differences between models (*P-value* < 0.01). Green lines are the theoretical maximum PAs.

The PAs ($r_{\hat{g}\;,p}$) of M2 to M5 in leave-one-year-location-out (CV1) and leave-one-breeding-cycle-out (CV2) are shown in Figure 4 for grain yield and Figure 5 for protein content.

The PA in CV1 varied from 0.296 to 0.528 for grain yield and 0.463 to 0.601 for protein content. A significant improvement in PA was observed for using predictions of the main additive effect plus the genomic [SNPs] × ECs interaction effect (**g** + **g*w***) compared to using only predictions from the main additive effect (**g**). The inclusion of the **g*w*** effect represented a significant increase of 16.4% for grain yield and 7.1% for protein content (M3 compared to M2). The MT models performing TA-GP (M4 and M5) showed a significant improvement in PA compared to the ST models and MT models without TA-PA. The TA-GP showed a significant PA increase of 37.9% for grain yield and 17.2% for protein content (M5 compared to M3). The highest PAs were obtained for both traits when combined TA-GP and G×E modeling through the genomic [SNPs] × ECs interaction effect in M5 (grain yield PA: 0.528, protein content PA: 0.601).

In CV2, the PA showed similar trends as CV1. A significant PA improvement was observed for models using the **g** + *g**w*** predictions compared to models using only predictions from the *g* effect. Using **g** + **g*w*** predictions represented a significant increase of 40.2% for grain yield and 14.1% for protein content (M3 compared to M2). The TA-GP for MT models provided a significant increase compared to the ST models and MT models without TA-PA, revealing a PA increase of 94.8% for grain yield and 59.7% for protein content (M5 compared to M3). The highest PA was obtained for both traits when TA-GP and G×E modeling through the genomic [SNPs] × ECs interaction effect in M5 (grain yield PA: 0.448, protein content PA: 0.452). In comparison, the CV2 had lower PA than CV1 for both traits, and higher benefits were observed in CV2 for modeling G×E and using TA-GP.

The maximum potential PA followed a similar trend for both traits, and the highest values were observed for the models accounting for genomic [SNPs] × ECs predictions (M3 and M5). The M1 (baseline) was not included in this section because such a model has no PA in CVs due to independence between lines is assumed.

### Variance inflation analysis and model validation

The estimates for variance inflation (*b*_*w,p*_) of the predicted genetic effects (Legarra and Reverter, 2018) for CV1 and CV2 are shown in Table 7 for grain yield and Table 8 for protein content.

**TABLE 7:** Slope of regression (*b*_*w,p*_) of estimated genetic values with whole information on genetic values with partial information for grain yield in leave-one-year-location-out and leave-one-breeding-cycle-out cross-validations.

Models	CV1: leave-one-year-location-out		CV2: leave-one-breeding-cycle-out	
	*g* [main effect]	*gw* [interaction effect]	*g* [main effect]	*gw* [interaction effect]
M2	1.02	–	0.80	–
M4	1.02	–	0.80	–
M4-TA	1.01	–	0.88	–
M3	1.02	0.87	0.89	0.79
M5	1.03	0.85	0.87	0.79
M5-TA	1.02	0.93	0.92	0.98

M2: line + genomic [SNPs] additive effect + spatial effect + line × environment interaction. M3 expand M2 by adding a genomic [SNPs] additive × environmental covariates (ECs) interaction. M4 expand M2 to the multi-trait case. M4-TA is the M4 using trait-assisted (TA) genomic prediction. M5 expand M3 to the multi-trait case. M5-TA is the M4 using TA genomic prediction. g: genomic [SNPs] additive effect. *gw*: genomic [SNPs] additive × ECs interaction effect.

**TABLE 8:** Slope of regression (*b*_*w,p*_) of estimated genetic values with whole information on genetic values with partial information for protein content in leave-one-year-location-out and leave-one-breeding-cycle-out cross-validations.

Models	CV1: leave-one-year-location-out		CV2: leave-one-breeding-cycle-out	
	*g* [main effect]	*gw* [interaction effect]	*g* [main effect]	*gw* [interaction effect]
M2	1.04	–	0.80	–
M4	1.03	–	0.81	–
M4-TA	1.03	–	0.88	–
M3	1.04	0.98	0.89	0.86
M5	1.04	0.95	0.90	0.81
M5-TA	1.04	1.00	0.93	0.98

M2: line + genomic [SNPs] additive effect + spatial effect + line × environment interaction. M3 expand M2 by adding a genomic [SNPs] additive × environmental covariates (ECs) interaction. M4 expand M2 to the multi-trait case. M4-TA is the M4 using trait-assisted (TA) genomic prediction. M5 expand M3 to the multi-trait case. M5-TA is the M4 using TA genomic prediction. g: genomic [SNPs] additive effect. *gw*: genomic [SNPs] additive × ECs interaction.

Predictions for the main additive effect (**g**) did not present variance inflation in CV1 since *b*_*w,p*_ values were close to one for both analyzed traits. The *b*_*w,p*_ for predictions of the genomic [SNPs] × ECs interaction effect (**g*w***) indicated a moderate over-dispersion for M3 (*b*_*w,p*_ = 0.87) and M5 (*b*_*w,p*_ = 0.85, without TA-GP) for grain yield. The use of TA-GP resulted in a reduction of over-dispersion in predictions of the **g*w*** effect for grain yield (M5-TA-GP: *b*_*w,p*_ = 0.92). For protein content the *b*_*w,p*_ values for predictions of the **g*w*** effect varied from 0.92 to 1.0, revealing lower over-dispersion than grain yield.

CV2 had higher variance inflation of predicted values than CV1, and trends were similar for both analyzed traits. The predictions of the main additive effect (**g**) presented the highest variance inflation for M2 (grain yield: *b*_*w,p*_ = 0.80, protein content *b*_*w,p*_ = 0.80) and the MT model M4 without TA-GP (grain yield: *b*_*w,p*_ = 0.80, protein content *b*_*w,p*_ = 0.81). A systematic improvement in the variance inflation for predictions of the main additive effect (**g**) was observed for both traits when the genomic [SNPs] × ECs interaction was included in the models (*b*_*w,p*_ from 0.80 in M2 to 0.89 in M3 for both traits). The *b*_*w,p*_ for predictions of the genomic [SNPs] × ECs interaction effect (**g*w***) varied from 0.79 to 0.98 for grain yield and 0.86 to 0.98 for protein content, where in general, the lowest values were associated in both traits to M3 and the MT model M4 without TA-GP, and the highest *b*_*w,p*_ to the M5 using TA-GP.

## Discussion

In this study, we proposed a growth degree-day (GDD) based reaction norm (RN) methodology to introduce genomic [SNPs] × environmental covariates (ECs) interactions *via* (co)variance structures in single-trait (ST) and multi-trait (MT) frameworks. The developed models were used for VCs estimation and genomic prediction of grain yield and protein content in a large set of advanced wheat breeding lines from the commercial company Nordic Seed A/S. The proposed models were evaluated using cross-validation (CV) analysis and trait-assisted genomic prediction (TA-GP) in two prediction problems relevant for plant breeding: (i) predicting breeding values for lines that have been tested in some environments but not in others (CV1, leave-one-year-location-out), and (ii) predicting breeding values for new lines across breeding cycles (CV2, leave-one-breeding-cycle-out). In CV1, the RN developed models have the potential to predict in environments where no lines have been tested, and thus, it could be used either to retrieve information from lines whose phenotyping has failed in a target environment or to predict for a completely new environment where no phenotyping has been performed before. Combining the proposed RN methodology and TA-GP proved to be an efficient approach to improve the predictive ability (PA) and reduce variance inflation of grain yield and protein content predictions in both CVs.

### Variance components

The VCs were estimated for the different models in ST and MT scenarios for grain yield (Table 4) and protein content (Table 5). A significant proportion of genetic variance was captured for both traits, as it can be observed in the narrow-sense (*h*^2^) and broad-sense (*H*^2^) heritabilities for M2. In agreement with previous studies using Nordic Seed A/S data, higher heritability for protein content than grain yield was observed (Kristensen et al., 2018; Kristensen et al., 2019; Guo et al., 2020; Raffo et al., 2022). The M2 was extended in order to better account for G×E interactions, and the genomic [SNPs] × ECs interaction term (**g*w***) was included in M3. The **g*w*** effect captured a significant proportion of variance for grain yield, and models including the G×E interaction terms **f (**line × environment) and **g*w*** (M3 and M5) captured higher proportion of G×E variance than models only including the **f** effect (M1, M2 and M4). The higher G×E variance captured by M3 and M5 can be likely attributed to better accounting for genetic and environmental covariances between lines. The trends were similar for protein content, but the variance for G×E interaction was considerably lower than for grain yield. As reported in previous studies (Jarquin et al., 2014; Pérez-Rodríguez et al., 2015; De Los Campos et al., 2020), the modeling of **g*w*** explain a limited proportion of G×E variance. It can happen due to SNPs and ECs not fully capturing the additive genetic effects (due to incomplete LD of QTLs and SNPs) and the environmental variation (e.g., due to distance between weather stations and the test field), respectively. To deal with this issue, the line × environment interaction effect is defined in the models to account for the mentioned misspecifications. In addition, another model term specifying genomic [SNPs] × environment interactions have been considered in the models; however, no significant contribution was observed and data has not been displayed since such an effect does not have the capacity of performing predictions in CV1. The inclusion of an additional term defining the main effect of ECs has been used in previous studies (Jarquin et al., 2014; Pérez-Rodríguez et al., 2015; De Los Campos et al., 2020); nevertheless, such an effect was not possible to estimate in our case as the main environmental effect is already defined at the level of fixed effects. Lastly, a significant proportion of spatial variance ($\sigma_{s}^{2}$) was observed for both traits, revealing a high spatial variability in the experimental fields. Our proposed methodology for modeling spatial effects has the advantage of not assuming any gradient or specific pattern across the field, allowing for spatial heterogeneity in any direction. This can present an advantage compared to previous methodologies that use X and Y coordinates (row-columns) as covariates to model spatial variation, as they may assume a gradient in X and Y directions (Bernal-Vasquez et al., 2014; Cericola et al., 2017); however, although it can be practical given its simplicity, the spatial variation can generally vary randomly without following a gradient across the field. The estimate obtained for $\sigma_{s}^{2}$ of grain yield in our study was consistent with the observed for a similar dataset and spatial effect definition in Raffo et al. (2022), and it was higher than in Cericola et al. (2017), where spatial effects were modeled in a row-column setting for a subset of our data. The differences between studies could be, at least in part, attributed to the mentioned differences in model assumptions. Given the significant proportion observed for $\sigma_{s}^{2}$ in our study, the inclusion of spatial effects in selection models is justified as it may help to improve the model specification.

The MT models showed similar VCs estimates to ST models. In general, high negative between-trait correlations were observed for the different model effects (Table 6). From a breeding perspective, the negative genetic correlation between grain yield and protein content implies an unfavorable response in one trait when selecting on another (Falconer and Mackay, 1996). The negative genetic trait correlations can be due to pleiotropy or tight gene linkage disequilibrium (Chen and Lübberstedt, 2010). Understanding the genetic basis for the negative correlation can help to define an appropriate breeding program and avoid affecting long-term breeding prospects. Note that for pleiotropic gene effect it can be recommended to select against those genes, while for undesirable linkage between genes the strategy would aim to break the linkage through recombination (Falconer and Mackay, 1996; Lynch and Walsh, 1998).

### Genomic predictive ability

The PA of the proposed models (Figures 4, 5) was calculated as the correlation between the average value of lines in each year-location after correcting by fixed effects and the vector of predictions (prediction of **g** effect for M2 to M5; predictions of **g** + **g***w* effects for M3 and M5). The predictions for the main additive effect (**g**) had the lowest values of PAs, and they were significantly outperformed by the models accounting for the genomic [SNPs] × ECs interaction when the sum of **g** + **g***w* effects was used. In CV1, using the sum of **g** + **g***w* effects results in an increase of 16.4% for grain yield and 7.1% for protein content (comparing M3 to M2). Predictions for **g** + **g***w* in CV1 are year-location specific and cannot be generalized to future years. In CV2, similar trends to CV1 were observed, but the increments for including the **g***w* predictions were considerably higher than in CV1, representing a 40.2% and 14.1% increase for grain yield and protein content, respectively (comparing M3 to M2). As shown in Figures 4, 5, the PAs were higher for protein content than for grain yield. These results could be related to the underlying architecture of the traits (Momen et al., 2018) and the differences found in *h*^2^, where for the lower *h*^2^ values as in grain yield, lower PA is expected (Wimmer et al., 2013; Muranty et al., 2015).

The CV2 had lower PAs for all models. The decline in PA can be explained by the higher genetic/pedigree distance between the training and validation population in CV2 (Habier et al., 2007; Wolc et al., 2011); while in CV1, the same line can be present in different year-locations folds, and full sibs are included in the training and validation sets, for CV2, the replication of the same line or close relatives are not shared between training and validation sets. This fact can also be evidenced in the average genomic relationships between reference and validation sets for CV1 and CV2. For example, the average of genomic relationships for reference/validation sets in CV2 was −0.009 with a standard deviation (SD) of 0.002; the negative value of relationships can be interpreted as lines less related than average. Conversely, in CV1, lines from the same breeding cycle were included in reference/validation sets, and the average genomic relationship within breeding cycles was 0.055 (SD: 0.015). The positive value obtained for relationships within breeding cycles can be interpreted as lines more related than average. As reported by Habier et al. (2007), the decline in PA can result from the break-up of linkage disequilibrium between SNPs and QTL between the training and validation populations when relationships become more distant.

### Multi-trait and trait-assisted genomic prediction

The MT models were evaluated for GP with and without TA-GP. The PAs of MT models without TA-GP were similar to ST models for the two CVs and traits (Figures 4, 5). Similar results have been found for wheat (Lado et al., 2018; Kristensen et al., 2019), and other species (Jia and Jannink, 2012; Schulthess et al., 2016), and it has been associated with high trait complexity and low traits heritabilities (Jia and Jannink, 2012; Lado et al., 2018). Conversely, MT models with TA-GP significantly improve PA compared to ST models and MT models without TA-GP. The superior performance of MT models with TA-GP can be explained by the use of “borrowed” information from the trait with complete phenotypic records, which is possible due the use of the between-trait correlations. The highest benefits for TA-GP were observed for grain yield PA, where it significantly increased (*P-value* < 0.01) by 14.0% (M2 compared to M4-TA) and 37.9% (M3 compared to M5-TA) for CV1, and by 57.9% (M2 compared to M4-TA) and 94.8% (M3 compared to M5-TA) for CV2. For protein content, the benefits provided by TA-GP were lower but still significant (*P-value* < 0.01), increasing PA by 5.4% (M2 compared to M4-TA) and 14.3% (M3 compared to M5-TA) for CV1, and by 28.2% (M2 compared to M4-TA) and 59.7% (M3 compared to M5-TA) for CV2. As reported by Jia and Jannink (2012) and Guo et al. (2014), the highest benefits conferred by the MT approach are expected for low heritability traits when they are used together with high heritability traits. Accordingly, our study empirically addressed this issue by combining a low heritability trait as grain yield with an intermediate to high heritability trait as protein content in bivariate analysis. Our results were consistent with the literature, revealing the highest benefits for the lowest heritability trait in all scenarios. Another factor contributing to the large benefit observed in TA-GP is the substantial correlation observed between grain yield and protein content (from −0.404 to −0.687 for genetic and G×E effects, Table 6). High between-trait correlations are statistically useful since measurements of one trait can more informative on the genetic values of the other correlated traits (Henderson and Quaas, 1976; Schaeffer, 1984; Thompson and Meyer, 1986; Montesinos-Lopez et al., 2016).

### Inflation of variance

The variance inflation (*b*_*w,p*_) for prediction of the main additive (**g**) and the genomic [SNPs] × ECs (**g*w***) interaction effects in CV1 and CV2 are shown in Tables 7, 8. The CV1, did not present variance inflation for **g** predictions as expected due to the high information in the training population for the **g** effect. However, **g*w*** predictions revealed over-dispersion in CV1. A possible explanation for this can be associated with the design of CV1, where no phenotypes for the environment predicted are kept in the training population, and therefore, differences in the QTL effect/expression in the specific environment may not be captured by SNPs, resulting in inflation of the predicted effects. In CV2, over-dispersion was observed in **g** and **g***w* predictions for both traits. This was expected as the genetic relationships between training and validation population are low, and over-dispersion due to genomic erosion by recombination can occur (Dekkers et al., 2021). Despite this, as described in the “Result” section, the over-dispersion of **g** predictions was reduced when the **g***w* effect was included in the models. In addition, a reduction in the over-dispersion of **g** and **g***w* predictions was observed for both traits in the two CVs when TA-GP was used in MT models. The benefits of using TA-GP can be related to a reduction of genomic erosion since phenotypic records of the additional trait are complete, and multi-trait modeling allows to indirectly capture the genetic effects for the trait of interest *via* the between-trait genetic correlations. The reduction in variance inflation conferred by TA-GP is consistent with previous studies, as reported by Pollak et al. (1984); Jia and Jannink (2012), Hayes et al. (2017), and Lado et al. (2018).

### Final remarks and future prospects

The proposed GDD based methodology provides a simplified approach to incorporate environmental information into prediction models and assists in the implementation of multi-trait reaction norm models. Our study showed that the weather and soil information was efficiently exploited without explicitly linking it to specific critical crop periods, which might open an opportunity to work with traits and species where the relationships between the environmental conditions and resulting phenotype are not fully established. A future avenue for developing the proposed models could be exploiting the potentiality of reaction norms in predicting G×E for future years (e.g., using average weather or soil variables to infer future years). The developed models work under the Bayesian Ridge Regression (BRR) framework and assume Gaussian distribution for random effects, implying no specific QTLs or ECs with large effects. Further studies exploring alternative methodologies capable of performing differential shrinkage or variable selection for genetic and environmental effects is warranted.

## Conclusion

In this study, we proposed a growth degree-day (GDD) based reaction norm methodology to introduce genomic [SNPs] × environmental covariates interactions through (co)variance structures in a single-trait or multi-trait framework. The growth degree-day based methodology provides a simplified approach to introduce environmental information in prediction models and assists in the implementation of multi-trait reaction norm models. The modeling of genomic [SNPs] × environmental covariates interactions, and the use of trait-assisted genomic prediction in multi-trait models, significantly enhanced the predictive ability and reduced variance inflation in the predicted genetic values for grain yield and protein content in leave-one-year-location-out (CV1) and leave-one-breeding-cycle-out (CV2) cross-validations. The genotype by environment interaction modeling *via* genomic [SNPs] × environmental covariates interactions, combined with trait-assisted genomic prediction, boosted the benefits in predictive performance.

## Data availability statement

The original contributions presented in this study are publicly available. This data can be found here: https://doi.org/10.7910/DVN/WEXJWW.

## Author contributions

JJ, MR, PS, and JA: conceptualization. MR, JA, PS, and JO: data curation. MR: formal analysis. JJ, MR, JA, JO, and AJ: funding acquisition. MR and JJ: investigation, methodology, and project administration. JJ and PS: resources and supervision. MR: software, validation, visualization, and writing—original draft. JJ, PS, JA, JO, and AJ: writing—review and editing. All authors contributed to the article and approved the submitted version.
