# Supplementary material for: Integrating a growth degree-days based reaction norm methodology and multi-trait modeling for genomic prediction in wheat
Source: Front Plant Sci. 2022 Sep 2;13:939448. doi: 10.3389/fpls.2022.939448 (PMC9481302; doi:10.3389/fpls.2022.939448)
Supplement: Supplementary file 2 [file Data_Sheet_1.docx]

**Supplementary material 2**

In this material, we present a convergence analysis for the parameters estimation. The Monte Carlo standard errors (MCMC error) were computed for all the estimated variance components (VCs) and are shown in **Table** **S2** for grain yield and **Table S3** for protein content.

**Table 2S**. Monte Carlo standard errors (MCMC error) for grain yield variance component.

| **Models** | **Main effect** | | |  | **Interactions** | | **Res.** |
| --- | --- | --- | --- | --- | --- | --- | --- |
|  | $\boldsymbol{l}$ | $\boldsymbol{g}$ | $\boldsymbol{s}$ |  | $\boldsymbol{f}$ | $\boldsymbol{gw}$ |  |
| M1 (Baseline) | 0,00012 |  | 0,00006 |  | 0,00007 |  | 0,00003 |
| M2 | 0,00018 | 0,00022 | 0,00005 |  | 0,00008 |  | 0,00003 |
| M3 | 0,00017 | 0,00019 | 0,00006 |  | 0,00014 | 0,00020 | 0,00003 |
| M4 | 0,00019 | 0,00020 | 0,00007 |  | 0,00008 |  | 0,00004 |
| M5 | 0,00016 | 0,00018 | 0,00007 |  | 0,00132 | 0,00019 | 0,00004 |

$\boldsymbol{l}$ line, $\boldsymbol{g}$ genomic [SNPs] additive effect, $\boldsymbol{s}$ spatial effect, $\boldsymbol{f}$ line × environment interaction, $\boldsymbol{gw}$ genomic [SNPs] additive **×** ECs interaction, **Res.** residuals.

**Table 3S**. Monte Carlo standard errors (MCMC error) for protein content variance component.

| **Models** | **Main effect** | | |  | **Interactions** | | **Res.** |
| --- | --- | --- | --- | --- | --- | --- | --- |
|  | $\boldsymbol{l}$ | $\boldsymbol{g}$ | $\boldsymbol{s}$ |  | $\boldsymbol{f}$ | $\boldsymbol{gw}$ |  |
| M1 (Baseline) | 0,00006 |  | 0,00005 |  | 0,00003 |  | 0,00003 |
| M2 | 0,00010 | 0,00020 | 0,00005 |  | 0,00004 |  | 0,00003 |
| M3 | 0,00009 | 0,00019 | 0,00005 |  | 0,00007 | 0,00006 | 0,00003 |
| M4 | 0,00010 | 0,00020 | 0,00006 |  | 0,00004 |  | 0,00003 |
| M5 | 0,00011 | 0,00018 | 0,00005 |  | 0,00006 | 0,00006 | 0,00003 |

$\boldsymbol{l}$ line, $\boldsymbol{g}$ genomic [SNPs] additive effect, $\boldsymbol{s}$ spatial effect, $\boldsymbol{f}$ line × environment interaction, $\boldsymbol{gw}$ genomic [SNPs] additive **×** ECs interaction, **Res.** residuals.

The effective sample size (ESS) for each estimated parameter are presented in **Tables** **S4** and **S5** for grain yield and protein content, respectively.

**Table 4S.** Effective sample size of posterior Gibbs samples for grain yield.

| **Models** | **Main effect** | | |  | **Interactions** | | | **Res.** |
| --- | --- | --- | --- | --- | --- | --- | --- | --- |
|  | $\boldsymbol{l}$ | $\boldsymbol{g}$ | $\boldsymbol{s}$ |  | $\boldsymbol{f}$ | $\boldsymbol{ge}$ | $\boldsymbol{gw}$ |  |
| M1 (Baseline) | 2014 |  | 1496 |  | 2310 |  |  | 2095 |
| M2 | 683 | 531 | 1686 |  | 2139 |  |  | 1995 |
| M3 | 542 | 251 | 1591 |  | 605 |  | 540 | 1958 |
| M4 | 551 | 582 | 1594 |  | 2031 |  |  | 2063 |
| M5 | 875 | 477 | 1592 |  | 621 |  | 442 | 1977 |

$\boldsymbol{l}$ line, $\boldsymbol{g}$ genomic [SNPs] additive effect, $\boldsymbol{s}$ spatial effect, $\boldsymbol{f}$ line × environment interaction, $\boldsymbol{gw}$ genomic [SNPs] additive **×** ECs interaction, **Res.** residuals.

**Table 5S.** Effective sample size of posterior Gibbs samples for protein content.

| **Models** | **Main effect** | | |  | **Interactions** | | | **Res.** |
| --- | --- | --- | --- | --- | --- | --- | --- | --- |
|  | $\boldsymbol{l}$ | $\boldsymbol{g}$ | $\boldsymbol{s}$ |  | $\boldsymbol{f}$ | $\boldsymbol{ge}$ | $\boldsymbol{gw}$ |  |
| M1 (Baseline) | 2977 |  | 1485 |  | 2016 |  |  | 1987 |
| M2 | 633 | 581 | 1564 |  | 2104 |  |  | 2055 |
| M3 | 849 | 526 | 1491 |  | 636 |  | 584 | 1629 |
| M4 | 667 | 607 | 1453 |  | 1521 |  |  | 1816 |
| M5 | 719 | 484 | 1475 |  | 781 |  | 556 | 2042 |

$\boldsymbol{l}$ line, $\boldsymbol{g}$ genomic [SNPs] additive effect, $\boldsymbol{s}$ spatial effect, $\boldsymbol{f}$ line × environment interaction, $\boldsymbol{gw}$ genomic [SNPs] additive **×** ECs interaction, **Res.** residuals.

Figure **S1** and **S2** show the trace plot of the Markov chains and posterior sample density for grain yield of model 5 (the more complex model in terms of number of effects and traits included).


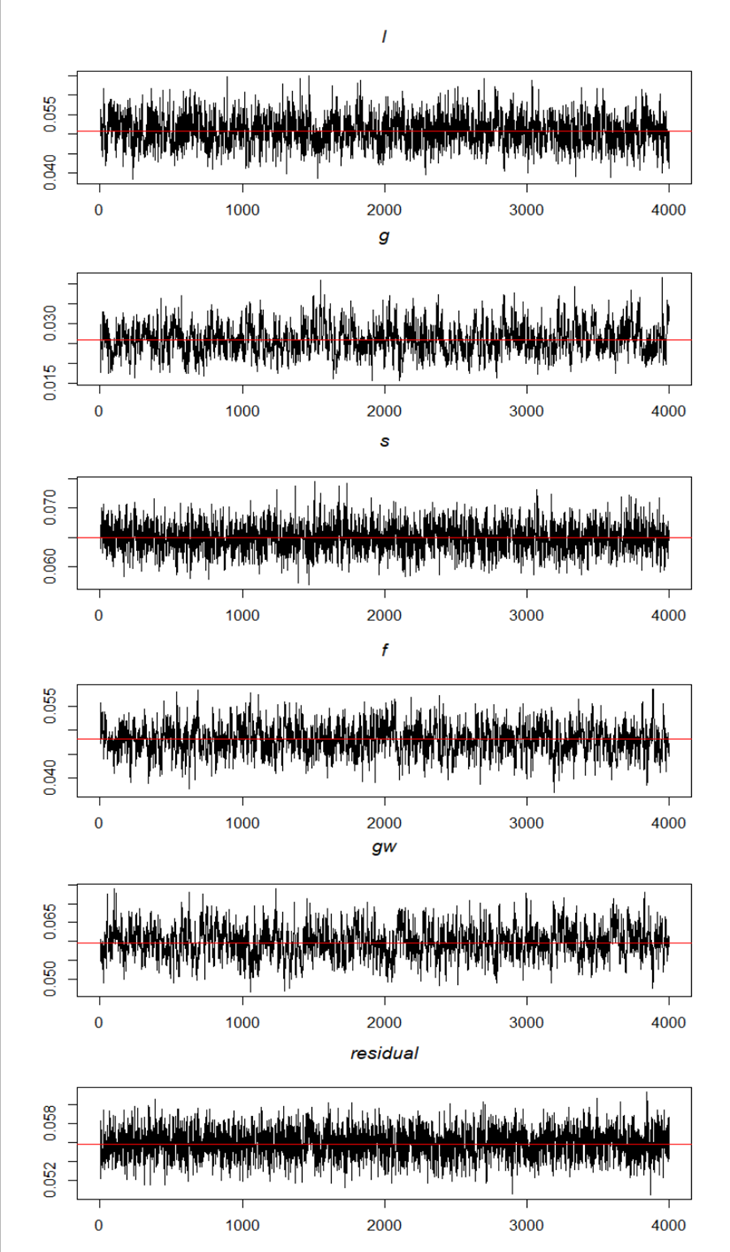


**Figure S1.** Trace plot of the Markov chains for grain yield in Model 5 (iterations number: 50.000, burn-in: 10,000, thinning: 10). $\boldsymbol{l}$ line, $\boldsymbol{g}$ genomic [SNPs] additive effect, $\boldsymbol{s}$ spatial effect, $\boldsymbol{f}$ line × environment interaction, $\boldsymbol{gw}$ genomic [SNPs] additive **×** ECs interaction. Red lines represent the mean estimated value.


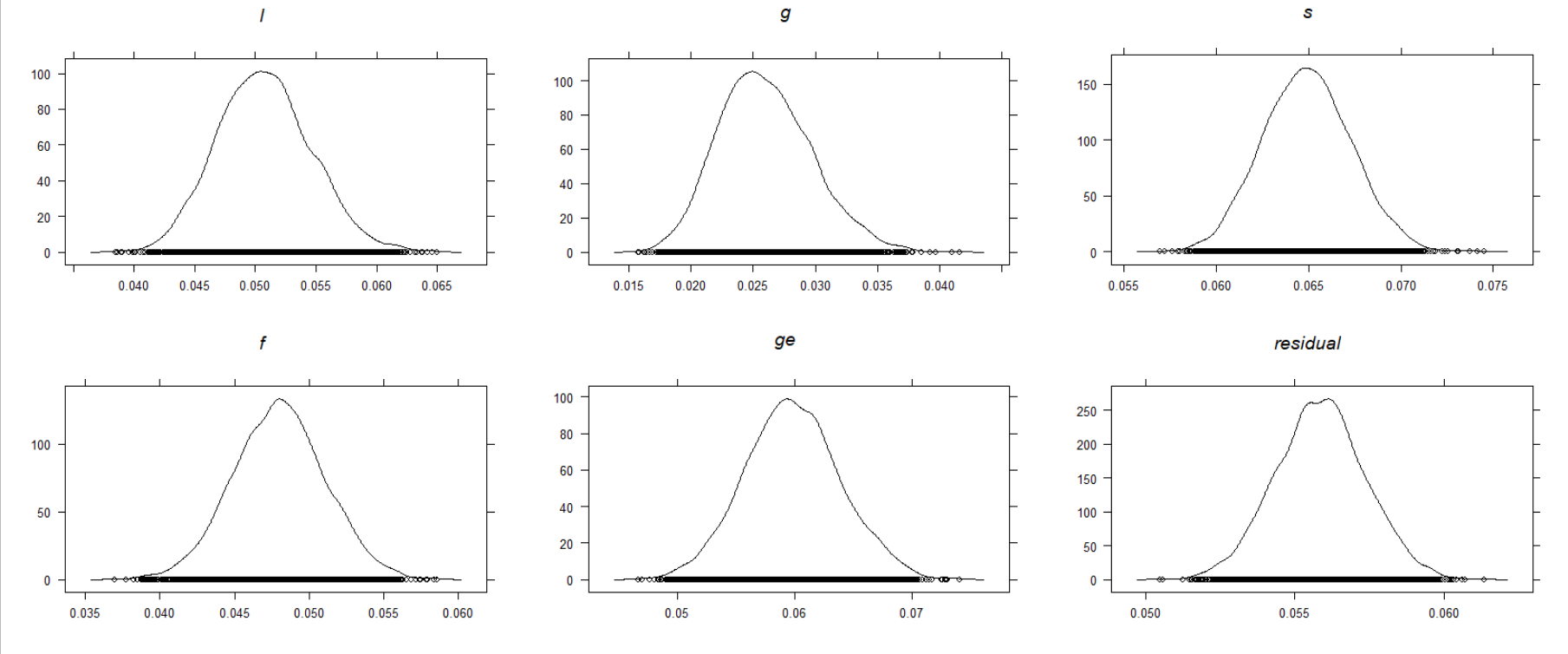


**Figure S2.** Posterior sample density of parameters for grain yield in model 5 (iteration number: 50.000, burn-in: 10,000, thinning: 10). $\boldsymbol{l}$ line, $\boldsymbol{g}$ genomic [SNPs] additive effect, $\boldsymbol{s}$ spatial effect, $\boldsymbol{f}$ line × environment interaction, $\boldsymbol{gw}$ genomic [SNPs] additive **×** ECs interaction.

Figure **S3** and **S4** show the trace plot of the Markov chains and posterior density of estimates for protein content of Model 5.


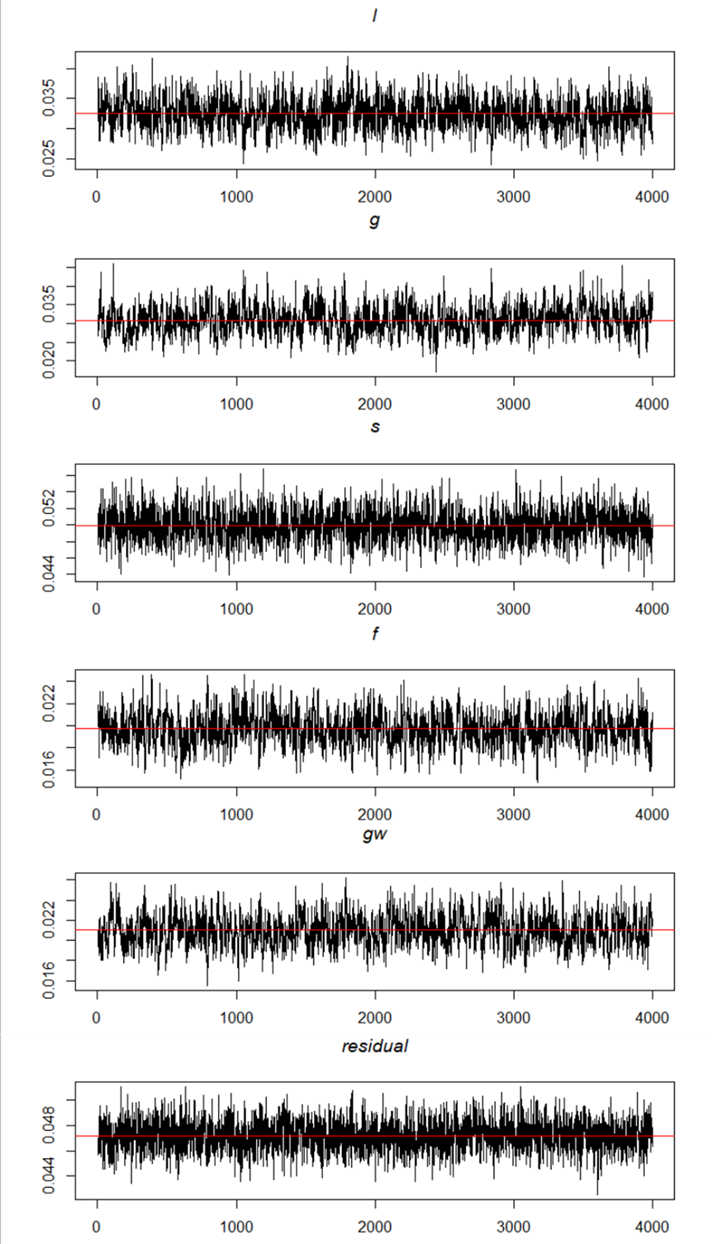


**Figure S3.** Trace plot of the Markov chains for protein content in Model 5 (iteration number: 50.000, burn-in: 10,000, thinning: 10). $\boldsymbol{l}$ line, $\boldsymbol{g}$ genomic [SNPs] additive effect, $\boldsymbol{s}$ spatial effect, $\boldsymbol{f}$ line × environment interaction, $\boldsymbol{gw}$ genomic [SNPs] additive **×** ECs interaction. Red lines represent the mean estimated value.


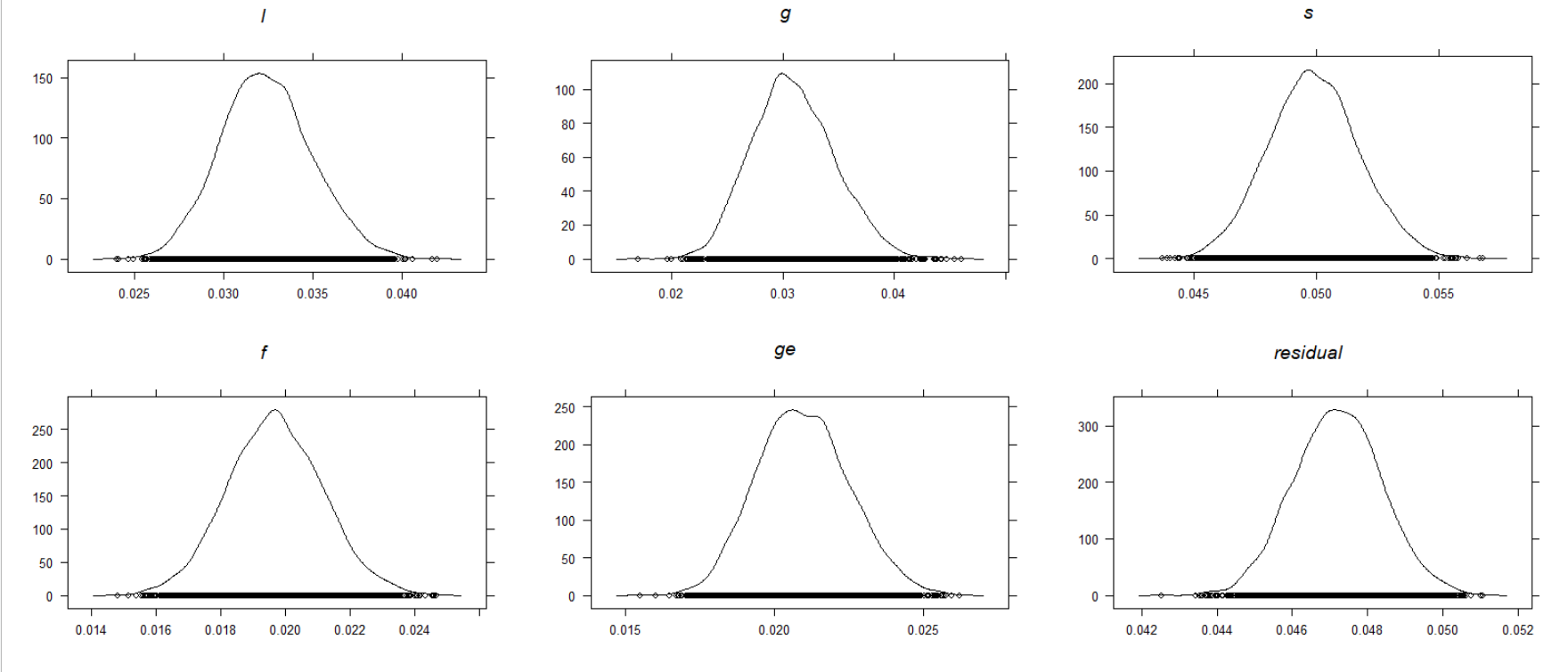


**Figure S2.** Posterior sample density of parameters for protein content in model 5 (iteration number: 50.000, burn-in: 10,000, thinning: 10). $\boldsymbol{l}$ line, $\boldsymbol{g}$ genomic [SNPs] additive effect, $\boldsymbol{s}$ spatial effect, $\boldsymbol{f}$ line × environment interaction, $\boldsymbol{gw}$ genomic [SNPs] additive **×** ECs interaction.
