# Supplementary material for: Integrating a growth degree-days based reaction norm methodology and multi-trait modeling for genomic prediction in wheat
Source: Front Plant Sci. 2022 Sep 2;13:939448. doi: 10.3389/fpls.2022.939448 (PMC9481302; doi:10.3389/fpls.2022.939448)
Supplement: Supplementary file 3 [file Data_Sheet_2.docx]

**Supplementary material 3**

This material presents schematic representations for leave-one-year-location-out cross-validation (CV1, **Figure S5**) and leave-one-breeding-cycle-out cross-validation (CV2, **Figure S6**).


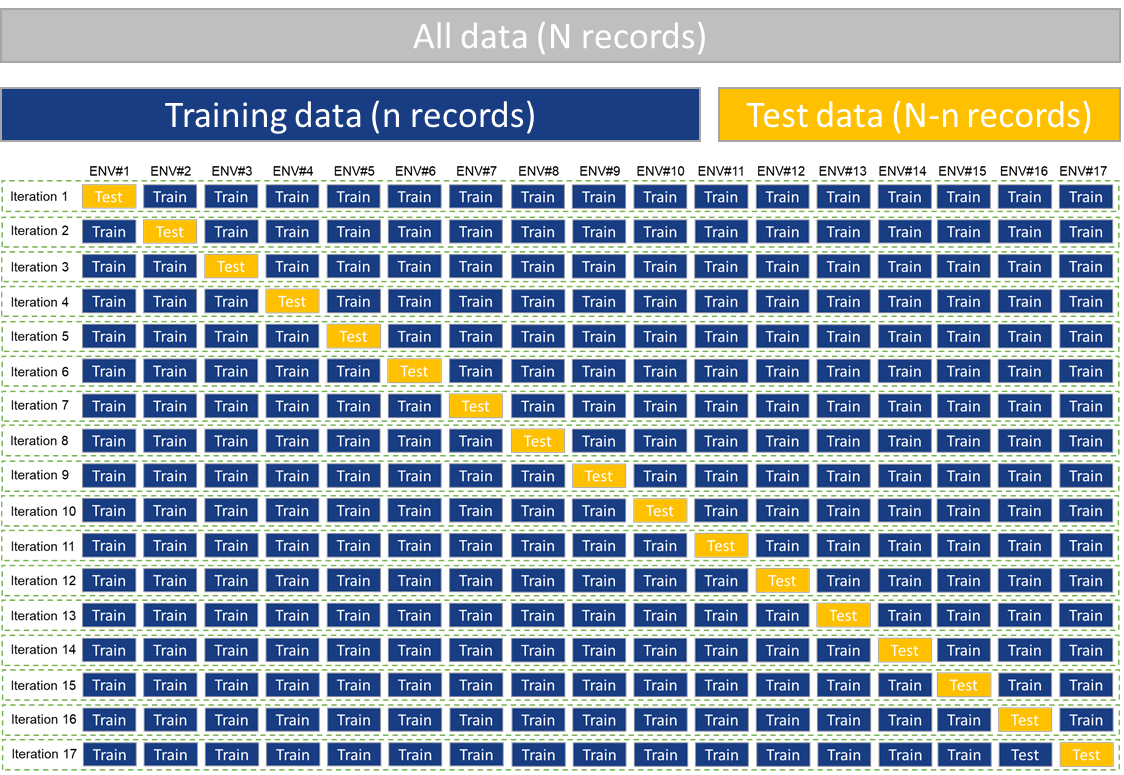


**Figure S5.** Schematic representation of leave-one-year-location-out (CV1). ENV#1 to ENV#17 represent the different environment (year-locarion combinations) where lines were growth.


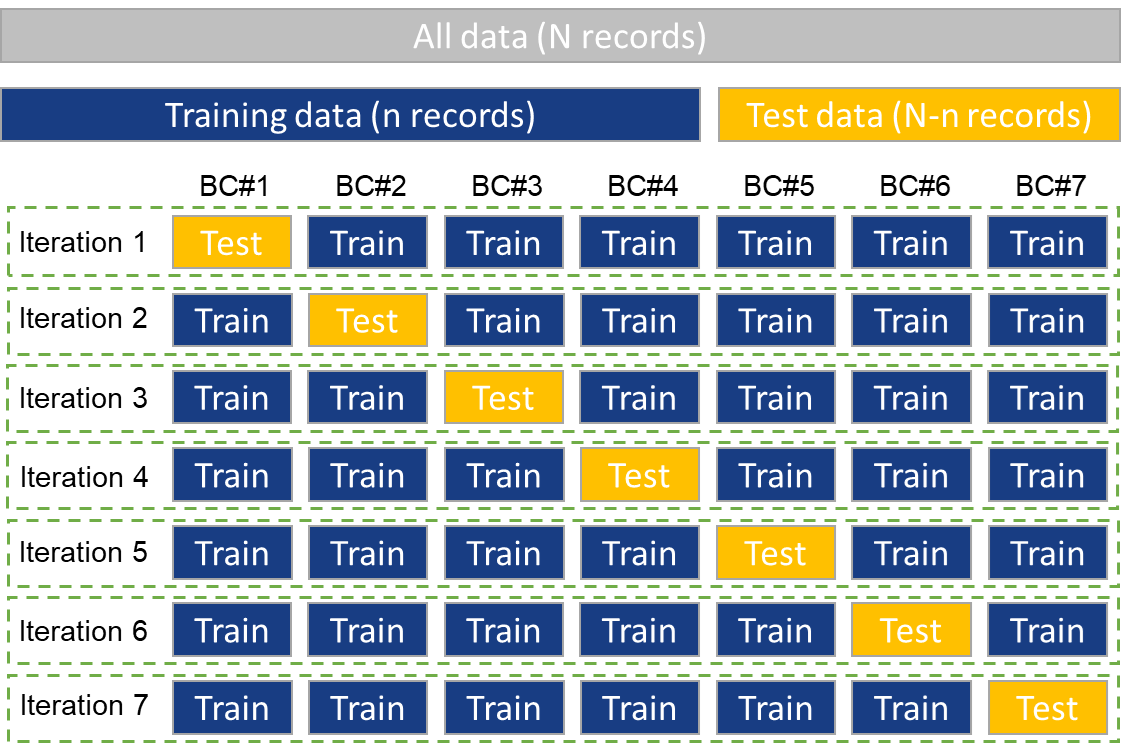


**Figure S6.** Schematic representation of leave-one-breeding-cycle-out (CV2). BC#1 to BC#7 represent the different F_6_ population coming from each of the seven different breeding cycles (BC).
